# Case Report: First pulmonary infection caused by *Mycobacterium colombiense* in a non-immunosuppressed host with bronchiectasis: diagnosis facilitated by synergistic mNGS and culture

**DOI:** 10.3389/fmed.2025.1671968

**Published:** 2025-10-30

**Authors:** Jishan Tan, Lu Liu, Lu Wang, Yuanqing Qu, Zhiyong Sun, Qin Wang, Yuan Liu

**Affiliations:** ^1^Department of Clinical Laboratory, The General Hospital of Western Theater Command, Chengdu, Sichuan, China; ^2^Department of Nuclear Medicine, The General Hospital of Western Theater Command, Chengdu, Sichuan, China

**Keywords:** *Mycobacterium colombiense*, non-immunosuppressed host, mNGS, bronchiectasis, non-tuberculous mycobacteria, NTM

## Abstract

*Mycobacterium colombiense*, a rare slow-growing mycobacterium within the *Mycobacterium avium complex* (MAC), causes disseminated disease almost exclusively in immunocompromised hosts, with no prior reports of localized pulmonary infection in non-immunosuppressed individuals. A 47-year-old non-immunosuppressed male with bronchiectasis presented with progressive cough, night sweats, and fatigue. Computed tomography (CT) revealed bronchiectasis with nodules in the right middle and lower lobes. Empirical β-lactam therapy failed, and conventional bronchoalveolar lavage fluid (BALF) tests (smears, cultures, PCR) yielded no pathogens at 48 h. Although metagenomic next-generation sequencing (mNGS) of BALF detected a low number of *M. colombiense* sequences (eight reads), definitive confirmation was achieved through extended culture, which is considered the gold standard for the diagnosis of nontuberculous mycobacteria. This culture revealed acid-fast bacilli within 12 days (160 CFU), confirming the presence of viable *M. colombiense*. Subsequent mNGS of the isolated colonies further confirmed the species identity with high sequence reads (25,787 reads). Guideline-based triple therapy (guided by drug susceptibility testing and guidelines) with clarithromycin, rifampicin, and ethambutol achieved significant radiographic resolution at 24 weeks. This case demonstrates that *M. colombiense* pulmonary infection is diagnostically elusive and mimics non-specific respiratory syndromes. It defines the clinical features of this pathogen in non-immunosuppressed hosts and highlights the need for heightened surveillance for nontuberculous mycobacteria (NTM) in bronchiectasis patients, given the likelihood of underdiagnosis.

## Introduction

Nontuberculous mycobacteria (NTM), encompassing all species beyond the *Mycobacterium tuberculosis* complex (MTBC) and *Mycobacterium leprae*, are environmentally ubiquitous organisms. Over 200 species have been identified to date (1), which act as opportunistic pathogens particularly threatening immunocompromised individuals, such as those with HIV infection (2–4) or organ transplant recipients (5). Although NTM can cause skin, lymph node, or disseminated infections, the lungs are the most common site of disease (6). NTM pulmonary disease (NTM-PD) presents a significant diagnostic challenge due to its non-specific symptoms and the difficulty in distinguishing true infection from colonization. Definitive diagnosis requires meeting clinical, microbiological, and radiological criteria as outlined in major international guidelines (7, 8). However, the low sensitivity and long turnaround time of traditional cultures frequently lead to misdiagnosis as tuberculosis or malignancy, delaying appropriate treatment (9, 10).

This diagnostic difficulty is compounded by a rising global incidence of NTM-PD, which has become a growing public health concern (11–14). The situation in China further illustrates this challenge, with a prevalence of 4.66%−6.3% for NTM infections among suspected tuberculosis cases (15, 16). The burden is higher in the south (up to 8.6%), where rapidly growing mycobacteria (RGM) dominate, unlike the north where the slowly growing *Mycobacterium avium* complex (MAC) is more common (15). The most prevalent species are *M. intracellulare* (40.5%) and *M. abscessus* (28.4%) (17), and immunocompromised hosts like HIV/AIDS patients bear a particularly high burden, with infection rates of 40%−46.7% largely due to MAC (18, 19). This complex epidemiology calls for heightened vigilance (15, 16, 20).

*M. colombiense* is a slow-growing NTM first isolated in 2006 from blood and sputum samples of HIV-positive patients in Colombia (21). It was subsequently identified as a distinct member of MAC (22). MAC is a clinically significant group of environmentally ubiquitous NTMs that includes species such as *M. avium* and *M. intracellulare* and is known to cause opportunistic infections primarily in immunocompromised individuals or those with underlying lung conditions (23). However, isolation of *M. colombiense* from clinical samples is uncommon. Existing research on this pathogen has predominantly focused on immunodeficient populations, with very limited evidence regarding its pathogenicity in non-immunosuppressed hosts.

Against this background, we present the first documented case of *M. colombiense* pulmonary infection in a non-immunosuppressed individual with bronchiectasis. A definitive diagnosis was established through standard culture procedures, which remain the cornerstone of NTM identification. Although mNGS provided rapid preliminary detection, culture isolation was critical for confirming *M. colombiense* as the causative agent. Subsequent drug susceptibility testing (DST) guided targeted antimicrobial therapy, facilitating prompt and effective treatment.

## Case presentation

On August 30, 2024, a 47-year-old male healthcare worker presented to our Infectious Diseases and Respiratory Department with a three-month history of persistent cough, night sweats, and progressive fatigue. He expressed particular concern about the worsening fatigue, which was impairing his daily work, and the night sweats, as he was troubled by the possibility of a protracted respiratory infection or other underlying systemic illness. His symptoms had an insidious onset and gradually worsened. Although a previous episode of acute sinusitis two months earlier had resolved, the cough, night sweats, and fatigue persisted. The patient denied any history of chronic diseases, immunodeficiency, exposure to endemic diseases, genetic risks, or known immunosuppression.

Physical examination was notable for the following findings. Vital signs were normal. The patient was alert and oriented with a lean build (BMI 20.60 kg/m^2^). On auscultation, scattered moist rales were audible in the bilateral lower lung fields, though no pleural friction rub was detected. Cardiovascular examination revealed normal heart sounds with a regular rhythm and no murmurs.

Laboratory investigations were conducted to assess the patient's systemic and immune status. Results of the complete blood count, liver and kidney function tests were all within normal ranges. Similarly, immune markers (T-cell subsets and immunoglobulins) and inflammatory markers were unremarkable. Serologic screening for HIV, syphilis, and autoimmune diseases returned negative results.

**Figure 1:**
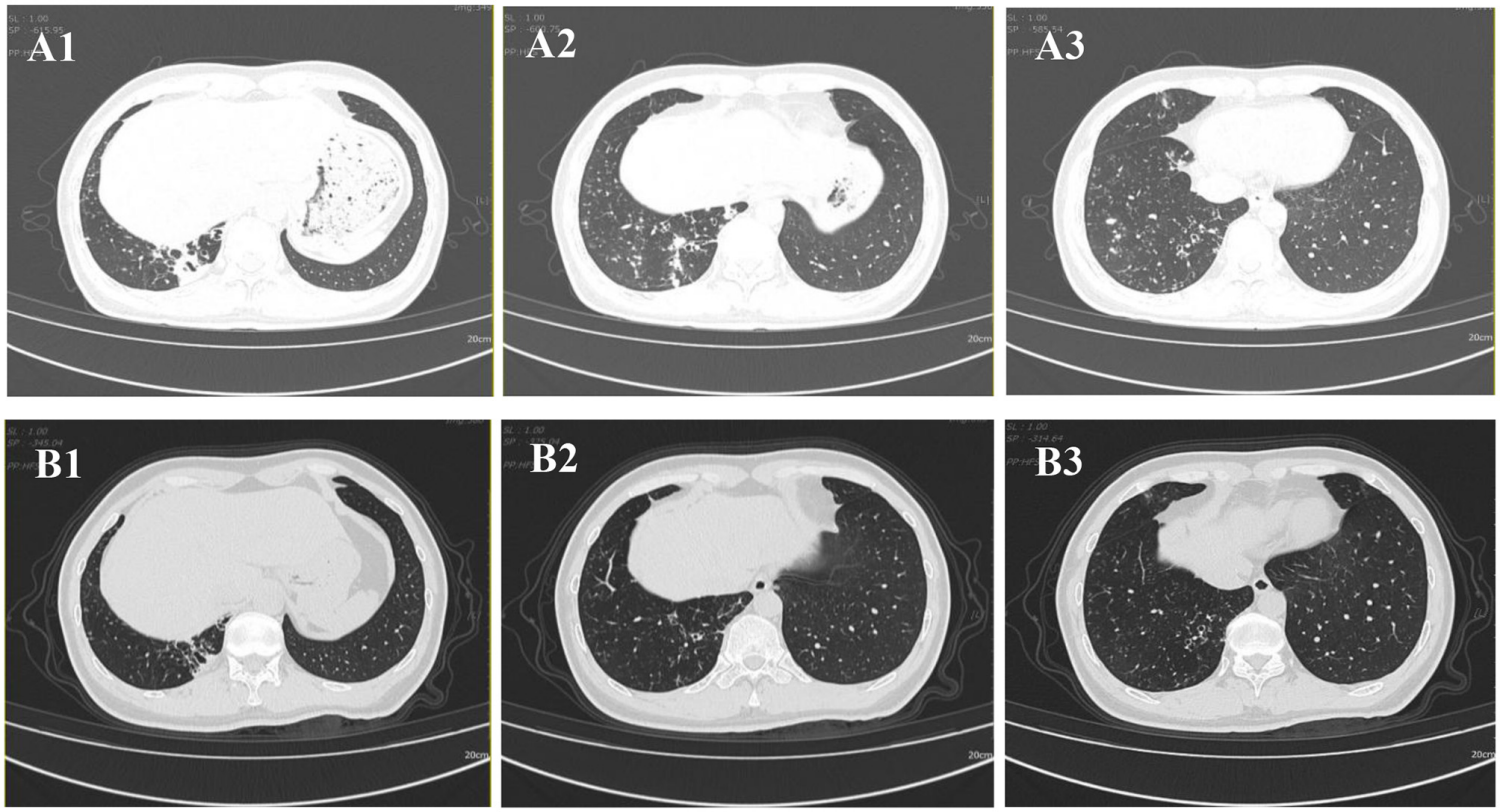
Chest CT images. **(A1–A3)** CT scan obtained on 2024-08-30 demonstrates bronchiectasis in the medial segment of the right middle lobe and the right lower lobe, accompanied by surrounding multiple patchy and nodular opacities. **(B1–B3)** Follow-up CT scan obtained on 2025-02-08 shows interval resolution of the peribronchial infectious foci in the right lower lobe.

Chest CT imaging, however, demonstrated significant pathology, revealing bronchiectasis in the medial segment of the right middle lobe and the right lower lobe. These areas were accompanied by multiple surrounding patchy opacities and nodules, suggestive of bronchiectasis with superimposed infection (Figures 1 A1–A3).

Consequently, the initial diagnosis was bronchiectasis with infection. To identify the causative pathogen, a sputum sample was obtained and sent for microbiological analysis. While microscopy revealed 1± Gram-positive cocci, the 48-h culture only yielded normal respiratory microbiota. Initial management included rest, nutritional support, antitussive medication (dextromethorphan), and empirical antibiotic therapy with piperacillin/tazobactam. However, by day 11 of treatment (September 10, 2024), the patient's symptoms showed no improvement and were progressively worsening. Fiberoptic bronchoscopy was subsequently performed. Analysis of BALF revealed: (1) Smear microscopy (Ziehl-Neelsen staining and Gram stain): negative; (2) Culture at 48 h: no bacterial or fungal growth; (3) Fluorescent PCR for *Mycobacterium tuberculosis* DNA: negative. This test was performed using a commercial kit (Singclean Medical, China) for nucleic acid extraction and PCR amplification (Mycobacterium tuberculosis complex nucleic acid detection kit) on a real-time PCR system (TIANKONG Gentier 96E, China). These results preliminarily excluded common bacterial, fungal, and tuberculous infections.

**Figure 2:**
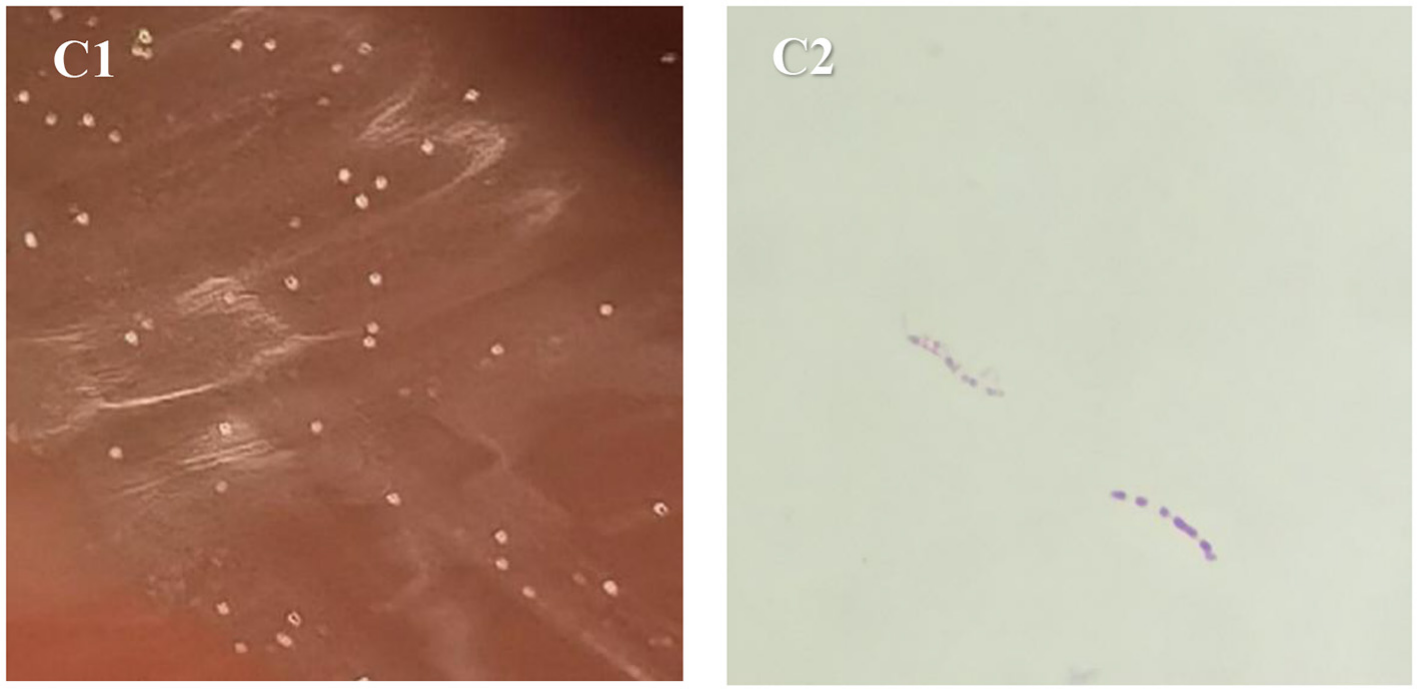
Morphological characteristics of *M. colombiense*. **(C1)** Culture results of blood agar on 2024-09-22; **(C2)** acid-fast-stained bacteria on 2024-09-22.

On September 12, 2024, mNGS analysis of BALF detected nucleic acid sequences of *M. colombiense* (eight reads), suggesting a possible NTM infection. This molecular finding prompted the extension of an ongoing BALF culture that had been initiated earlier. The culture had been inoculated onto blood agar and incubated at 37 °C in a 5% CO_2_ incubator. Given that *M. colombiense* is a slow-growing mycobacterium, and encouraged by the mNGS signal, we decided to prolong the incubation period beyond the initial 48 h. To prevent desiccation and contamination, the gap between the lid and base of the Petri dish was sealed along the edge with transparent tape, and colony growth was observed every three days. After extending incubation to a total of 12 days (until September 22, 2024), pale yellow, umbonated colonies (approximately 0.2 mm in diameter; Figure 2 C1) became visible, with an estimated count of 160 CFU. Gram staining of these colonies yielded negative results, while acid-fast staining (Ziehl–Neelsen) confirmed the presence of short-chain acid-fast bacilli (Figure 2 C2).

However, two independent matrix-assisted laser desorption/ionization time-of-flight mass spectrometry (MALDI-TOF MS) analyses using the Clin-TOF II system (Bioyang, China) yielded inconsistent species identifications with low confidence scores: the first identified *Mycobacterium tuberculosis* (score 25), and the second identified *Mycobacterium bovid* (score 30). Both results showed poor reproducibility, confirming the unreliability of MS-based identification for this isolate.

To definitively identify the pathogen, mNGS was performed directly on isolated colonies. Sequencing was conducted on an Illumina Nextel 550AR instrument. Following host depletion, libraries were prepared from the extracted DNA using the NextEra XT DNA Library Preparation Kit (Illumina, USA) according to the manufacturer's instructions. Microbial sequences were aligned against a curated database of approximately 13,000 bacterial, fungal, and parasitic genomes using SNAP. This analysis yielded 25,787 sequences uniquely mapping to *M. colombiense*, confirming species-level identification.

**Figure 3:**
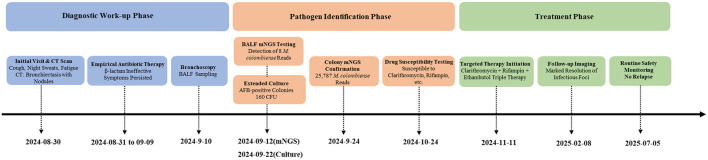
Diagnostic and therapeutic workflow of the case.

According to the Clinical Practice Guidelines for NTM-PD (7), diagnosis requires meeting all of the following criteria: (1) Pulmonary and/or systemic symptoms; (2) Radiographic findings consistent with NTM-PD (e.g., bronchiectasis with nodules on CT); (3) Exclusion of other pulmonary diseases; (4) Positive culture from at least one BALF sample. This patient fulfilled all criteria: CT showed bronchiectasis with infection, BALF culture confirmed *M. colombiense*, and symptoms persisted despite prior therapy. Therefore, he was definitively diagnosed with NTM-PD caused by *M. colombiense*. The diagnostic workflow that led from initial presentation to targeted therapy is outlined in Figure 3.

DST was performed on the isolated and purified strain using the broth microdilution method with a panel (Baso, Zhuhai, China), strictly according to the manufacturer's instructions. Results were interpreted following the criteria set forth in the Clinical and Laboratory Standards Institute (CLSI) guidelines M62, 1st ed and M24 3rd ed. Results indicated susceptibility to clarithromycin, rifampicin, rifabutin, and amikacin, intermediate susceptibility to doxycycline, and resistance to minocycline (Table 1).

**Table 1:** Susceptibility results and breakpoints for *M. colombiense*.

**Antibiotics**	**MIC (μg/mL)**	**Result**	**Breakpoints (**μ**g/mL)**		
			**Susceptible (S)**	**Intermediate (I)**	**Resistant (R)**
Amikacin	≤ 2	S	≤ 16	32	≥64
Clarithromycin	≤ 1	S	≤ 8	16	≥32
Linezolid	4	S	≤ 8	16	≥32
Moxifloxacin	≤ 0.25	S	≤ 1	2	≥4
Ciprofloxacin	1	S	≤ 1	2	≥4
Doxycycline	2	I	≤ 1	2–4	≥8
Minocycline	8	R	≤ 1	2–4	≥8
Rifabutin	≤ 0.5	S	≤ 2		≥4
Rifampin	0.5	S	≤ 1		≥2
Trimethoprim/Sulfamethoxazole	≤ 0.25/4.8	S	≤ 2/38		≥4/76
Amoxicillin/Clavulanate	≤ 4/2	S	≤ 8/4	16	≥32/16
Cefoxitin #	8	-	-	-	-
Imipenem #	4	-	-	-	-
Meropenem #	≤ 2	-	-	-	-
Tobramycin #	≤ 1	-	-	-	-
Tigecycline #	4	-	-	-	-

#No breakpoints available for these drugs.

As guidelines mandate treatment over observation for patients fulfilling the diagnostic criteria of NTM-PD with progressive symptoms (7), targeted therapy was commenced on November 11, 2024, due to the patient's clinical deterioration. The regimen consisted of clarithromycin (1.0 g daily), rifampicin (0.45 g daily), and ethambutol (0.75 g daily). Monthly complete blood counts and liver/kidney function tests were performed to monitor for adverse drug effects.

Following targeted therapy, the patient's clinical symptoms improved significantly. A follow-up chest CT on February 8, 2025 (Figure 1 B1–B3), showed marked resolution of the bronchiectasis with nodules compared to baseline (August 30, 2024). Subsequent follow-up at 24 weeks (until July 2025) confirmed sustained clinical stability without relapse and no reported drug-related adverse events.

## Discussion

This case provides the first documented evidence that pulmonary infection caused by *M. colombiense* can occur in non-immunosuppressed hosts, highlighting the pathogen's rare ability to breach the immune defenses of such individuals. Previous reports describe this bacterium almost exclusively affecting immunocompromised populations (e.g., HIV-positive individuals, organ transplant recipients), predominantly causing disseminated disease (involving ≥2 non-contiguous organ systems) (24). In stark contrast, our patient had no evidence of immunodeficiency, and the disease was confined to the lungs (bronchiectasis with infection in the right middle and lower lobes). This challenges the prevailing notion that “*M. colombiense* infection necessitates underlying systemic immunodeficiency.”

It is well-established that MAC infections are primarily acquired from environmental sources through inhalation, ingestion, or skin contact, manifesting as pulmonary disease, lymphadenitis, skin and soft tissue infections, or disseminated disease (7, 25–27). Risk factors for NTM-PD can be broadly categorized into three groups: host-related factors, drug-induced immunosuppression, and environmental exposure. Among these, host factors play a particularly critical role. Pre-existing pulmonary diseases—such as previous tuberculosis, bronchiectasis, chronic obstructive pulmonary disease (COPD), among others—significantly increase the risk of developing NTM-PD (23, 28–31). The underlying mechanism lies in the fact that these conditions often lead to impaired bronchial mucociliary clearance, thereby compromising local pulmonary defense. Recent studies further indicate that even in the absence of overt systemic disease or an immunosuppressed state, such localized impairment of mucociliary clearance alone can constitute an important risk factor for NTM infection (32–34). As an environmental mycobacterium, *M. colombiense* exploits defects in local host defenses to establish infection. In this case, although the host was not immunosuppressed, the presence of bronchiectasis likely facilitated *M. colombiense* infection by disrupting the mucosal barrier and compromising ciliary clearance. This highlights the critical need for heightened clinical vigilance for NTM infection in patients with structural lung diseases such as bronchiectasis, irrespective of their systemic immune status.

Cases of *M. colombiense* infection are rarely reported, likely due to historical diagnostic challenges in accurately distinguishing between MAC subspecies (3). The definitive diagnosis in this case relied on the synergistic application of mNGS and culture techniques. Although mNGS of BALF initially provided a crucial clue with a low sequence count (eight reads), it was insufficient for definitive diagnosis. Subsequent culture successfully isolated colonies within 12 days, a notably shorter duration than previously reported for respiratory specimens (4, 5, 9). Furthermore, MALDI-TOF MS failed to identify the species, likely due to the absence of reference spectra for *M. colombiense* in the database. Ultimately, definitive species-level identification was achieved by applying mNGS directly to the cultured isolate, which yielded 25,787 *M. colombiense* sequences. This synergistic approach of culture and mNGS was therefore critical for confirming the infection.

Drug therapy for NTM disease requires careful consideration due to the diversity of NTM species and their varying drug susceptibility profiles. Current guidelines strongly recommend performing *in vitro* DST before initiating treatment (35). The regimen should include at least two drugs likely or known to be effective against the specific NTM isolate; severe cases may require 4–6 effective drugs, and monotherapy must be avoided (36, 37). Our DST results demonstrated susceptibility to clarithromycin and rifampicin but resistance to minocycline. The subsequent successful clinical and radiological response to a DST-guided triple-drug regimen (clarithromycin, rifampicin, and ethambutol) further validates this approach (Figure 1 B1–B3 vs. Figure 1 A1–A3), and is consistent with treatment outcomes described in other susceptible cases (5, 24). However, a critical comparison with the broader literature reveals noteworthy heterogeneity in the drug susceptibility profiles of *M. colombiense*. Most notably, a contrasting case from Brazil involved an isolate exhibiting high-level resistance to both clarithromycin and rifampicin (9), which culminated in a fatal outcome despite therapy. This discrepancy underscores that susceptibility to first-line macrolides and rifamycins should not be universally assumed. Therefore, the variations in susceptibility profiles among *M. colombiense* isolates highlight the importance of DST (38). Guided treatment based on individual DST results remains essential for effective clinical management.

The management of suspected *M. colombiense* infection in resource-limited settings, where access to mNGS is unavailable, must rely on a structured, stepwise approach. Diagnosis should be pursued through sustained mycobacterial culture and acid-fast staining of respiratory specimens, which remain the fundamental and accessible methods for confirming NTM infection (39, 40). For treatment, initiating an empiric macrolide-based multidrug regimen following standard MAC guidelines is the cornerstone of management. The combination of clarithromycin, ethambutol, and a rifamycin is the recommended first-line therapy, with the addition of amikacin considered in severe or refractory cases (10, 41). While clarithromycin serves as the cornerstone with a relatively low resistance rate, the potential for emergent resistance necessitates monitoring, ideally via phenotypic DST when available (42, 43). In the absence of routine DST, therapeutic efficacy must be meticulously evaluated through clinical assessment and serial radiographic changes, with regular patient follow-up to prevent relapse (41, 44). This integrated strategy of conventional microbiology, guideline-based empiric therapy, and vigilant clinical-radiographic monitoring provides a viable framework for the effective management of *M. colombiense* infection where advanced resources are constrained.

Given these diagnostic hurdles, the incidence of *M. colombiense* is likely underestimated. Thus, in bronchiectasis patients with infection refractory to conventional antibiotics, NTM infection should be suspected and appropriate diagnostic tests pursued, even in non-immunosuppressed hosts. However, this study is limited by its nature as a single-case report. Therefore, it cannot provide a systematic assessment of the epidemiology, full spectrum of clinical manifestations, or long-term prognosis of *M. colombiense* infections in non-immunosuppressed hosts. Future multi-center, prospective studies are needed to better characterize this pathogen and optimize its clinical management.

## Conclusions

In conclusion, infections caused by *M. colombiense* are extremely rare. Compared to existing studies focusing on immunocompromised hosts, our case highlights that *M. colombiense* pulmonary infection can occur even in patients without demonstrable immunodeficiency. Therefore, both mNGS and optimized cultures are indispensable, serving as complementary diagnostic modalities for the effective identification and management of challenging NTM infections. Given the current limitations in diagnostic, surveillance, and reporting systems for *M. colombiense* infection, its true prevalence is likely underestimated, and the condition is prone to misdiagnosis. Clinicians should maintain vigilance for *M. colombiense* as a potential causative agent in bronchiectasis patients with refractory infections.

## Data Availability

The original contributions presented in the study are included in the article/supplementary material, further inquiries can be directed to the corresponding author.
